# An absence of equipoise: Examining surgeons’ decision talk during encounters with women considering breast cancer surgery

**DOI:** 10.1371/journal.pone.0260704

**Published:** 2021-12-16

**Authors:** Mary C. Politi, Catherine H. Saunders, Victoria F. Lenihan, Renata W. Yen, Amy E. Cyr, Marie-Anne Durand, Glyn Elwyn

**Affiliations:** 1 Department of Surgery, Division of Public Health Sciences, Washington University in St Louis School of Medicine, St Louis, MO, United States of America; 2 Dartmouth-Hitchcock Medical Center, The Dartmouth Institute for Health Policy and Clinical Practice, Lebanon, NH, United States of America; 3 The Johns Hopkins University School of Medicine, Baltimore, MD, United States of America; 4 Department of Medicine, Division of Medical Oncology, Washington University in St Louis School of Medicine, St Louis, MO, United States of America; 5 Centre d’Epidémiologie et de Recherche en santé des Populations, Université de Toulouse, INSERM UMR1295, Université Toulouse, Toulouse, France; 6 Unisanté, Centre universitaire de médecine générale et santé publique, Lausanne, Switzerland; Duquesne University, UNITED STATES

## Abstract

Shared decision-making is recommended for decisions with multiple reasonable options, yet clinicians often subtly or explicitly guide choices. Using purposive sampling, we performed a secondary analysis of 142 audio-recorded encounters between 13 surgeons and women eligible for breast-conserving surgery with radiation or mastectomy. We trained 9 surgeons in shared decision-making and provided them one of two conversation aids; 4 surgeons practiced as usual. Based on a published taxonomy of treatment recommendations (*pronouncements*, *suggestions*, *proposals*, *offers*, *assertions*), we examined how surgeons framed choices with patients. Many surgeons made *assertions* providing information and advice (usual care 71% vs. intervention 66%; p = 0.54). Some made strong *pronouncements* (usual care 51% vs. intervention 36%; p = .09). Few made *proposals* and *offers*, leaving the door open for deliberation (proposals usual care 21% vs. intervention 26%; p = 0.51; offers usual care 40% vs. intervention 40%; p = 0.98). Surgeons were significantly more likely to describe options as comparable when using a conversation aid, mentioning this in all intervention group encounters (usual care 64% vs. intervention 100%; p<0.001). Conversation aids can facilitate offers of comparable options, but other conversational actions can inhibit aspects of shared decision-making.

## Introduction

Shared decision-making is a process whereby clinicians work collaboratively with patients to reach an agreed-upon treatment plan that aligns with evidence and patients’ preferences [1]. Many clinicians report that they consistently engage in shared decision-making when there is more than one reasonable care option [2]. When asked about shared decision-making explicitly, most clinicians support the approach [2, 3]. In practice, clinicians often share information and allow patients to ask questions, but few present true clinical equipoise, fully explore patient preferences [2, 4], or promote patient-clinician collaboration [5]. Researchers have yet to thoroughly examine the ways in which clinicians might influence patients’ choices through explicit or subtle recommendations during consultations [6].

Stivers and Barnes [7] created a taxonomy of recommendations, categorized across five actions: *pronouncements*, *suggestions*, *proposals*, *offers*, *and assertions*. These actions represent a gradient of recommendations based on the strength with which they are communicated. *Pronouncements* are the most authoritative recommendations, delivered without seeking input from patients [7]. *Suggestions* follow in this taxonomy, where a clinician maintains relative agency over patients’ decision, but might inform patients that they have a role. *Proposals* occur when a clinician endorses collaboration with a patient, but offers recommendations during the process. *Offers* change the dynamics of the patient/clinician interaction. The clinician invites the patient to share input that aligns with patients’ preferences and values, and makes a recommendation based on those preferences. Finally, *assertions* provide broad information about options and their benefit. Clinicians can present these options as relatively equal in terms of outcomes or can use assertions to bias choices, depending on the tone and strength of the assertions. The taxonomy does not have a category which would align with a neutral stance, such as acknowledging clinical equipoise while discussing more than one reasonable choice, but overall provides a useful framework for understanding how clinicians might approach conversations about care decisions.

It is often difficult to isolate how clinicians engage in shared decision-making [8]. Some communication scholars suggest that fostering collaboration can be even more important to shared decision-making than communicating information and encouraging patients to weigh trade-offs between options [5]. In the *What Matters Most* trial [9, 10], surgeons randomized to one of two intervention groups were trained to use shared decision-making, discuss clinical equipoise, and support shared decision-making with one of two breast cancer surgery conversation aids. Surgeons randomized to usual care provided care as they typically did. Breast-conserving surgery with radiation and mastectomy offer equivalent breast cancer-specific outcomes for patients with early-stage breast cancer [11, 12], representing a preference-sensitive decision [13] appropriate for shared decision-making [1]. Each surgery involves different potential harms, benefits and long-term consequences but have similar survival benefit [13]. National organizations encourage surgeons to ensure that women understand that a more conservative surgery (breast-conserving surgery with radiation) is as likely to lead to survival as mastectomy; some women mistakenly think a larger surgery will improve survival [14–16]. Given an individual patient, though, a surgeon might not agree that there is true equipoise between the options because of the tradeoffs between them. The low annual recurrence rate following breast conservation accumulates and may seem unacceptably high over time for younger patients [17, 18]. In this situation, a surgeon might encourage mastectomy over breast conservation. Mastectomy with reconstruction could be preferred by a surgeon who, based on experience, expects a patient to have a poor cosmetic result with breast conservation [19]. Or, because a mastectomy is more invasive than breast conservation, and requires longer recovery (especially if a woman opts for breast reconstruction with a potential risk of perioperative complications [20, 21], breast conservation could be a better option for some patients with comorbidities or increased baseline operative risk [22, 23]. Similarly, because of recovery time or baseline operative risk, breast conservation could be preferred for a patient without a postoperative caretaker at home or who has limited access to a radiation facility or a plastic surgeon [24–26]. The *What Matters Most* trial provided an opportunity to examine how surgeons talk to women about this nuanced decision, while the trial provided tools and training in shared decision-making for those in the intervention groups.

Using a purposive sample of transcripts of audio-recorded encounters from the *What Matters Most* trial, this study aimed to:

1. Examine how surgeons talked to patients with early-stage breast cancer about surgery options, categorizing their approach using the Stivers and Barnes’ taxonomy of recommendations.
2. Compare their approach and use of recommendations between usual care and the intervention groups.

## Materials and methods

### Study design and data

This study involved a secondary analysis of data from the *What Matters Most* study, a comparative effectiveness trial of two conversation aids (called Option Grids) for women diagnosed with early-stage breast cancer. The study design and data are summarized below; see the study protocol and results for details [9, 10].

### Eligibility and screening

We recruited participants from September 2017 to February 2019. Patients’ inclusion criteria were: assigned female at birth; at least 18 years of age; diagnosis of early-stage breast cancer (stages I-IIIA); eligible for both breast-conserving surgery with radiation and mastectomy based on medical records, confirmed by the treating surgeon; spoke English, Spanish, or Mandarin Chinese. Exclusion criteria were: transgender men and women (transgender women who develop breast cancer have different baseline breast cancer risk that can affect surgery choice; some transgender women might be on hormones that impact breast tissue, cancer risk, and surgery choice; some transgender men are more likely to choose mastectomy as it could be a more gender-affirming option); previous prophylactic mastectomy; severe visual impairment, mental illness or dementia that would impact informed consent or study procedures; inflammatory breast carcinoma. Surgeons were eligible if they were an attending physician and performed breast surgery at one of the recruitment sites. We collected data at four National Cancer Institute-designated cancer centers in both urban and rural locations in the United States.

### Procedures

In the parent study, we randomized sixteen surgeons to one of three study groups: Picture Option Grid (conversation aid with shorter, simpler text and pictures in tabular format), Option Grid (conversation aid with text in tabular format) or usual care [9, 10]. Please see S1 Appendix for images. They were trained in person or by phone in the study protocol and use of the interventions to support shared decision-making, if assigned to the intervention groups. All surgeons, regardless of study arm, received training on the trial aims and procedures via a PowerPoint presentation delivered by the study PI (M-A.D.) or the study coordinator (R.W.Y.). Surgeons randomly assigned to an intervention arm received additional training on shared decision-making via the 3-talk model [27] (including how to describe clinical equipoise during Option Talk). They also received general information about conversation aids and didactic training on how to use their conversation aid, including a 3-minute video and role-play simulation. The study PI (M.-A.D.) delivered training on the use of the interventions to ensure uniformity across all surgeons. We delivered all but 2 of these trainings in person.

All surgeons agreed to have encounters recorded if patients consented. Audio-recorders were placed in exam rooms during all surgeon-patient encounters to reduce the impact of recording knowledge on surgeon behavior. We covered the recording light and placed the recorder face down; the recorder was only turned on with patient participants’ consent. A HIPAA-compliant service transcribed each recording verbatim.

Dartmouth College’s Committee for the Protection of Human Subjects (STUDY00030157), Washington University in St. Louis’ Institutional Review Board (201704011), and New York University’s Institutional Review Board (i17-00871) approved the study (the fourth institution had an institutional agreement with Dartmouth College’s Committee). We registered the trial on clinicaltrials.gov (NCT03136367). We obtained written consent from all participants.

### Data & analysis

#### Codebook.

We developed a codebook based on Stivers and Barnes’ taxonomy [7]. The full codebook is shown in S2 Appendix, and a description of each category of action is shown in Table 2. To capture the description of clinical equipoise, we added a category called “*offering comparable options*,” which could be done within several of Stivers and Barnes’ categories. We operationalized the codebook based on consensus and piloting.

#### Coding transcripts and analysis.

We used purposive sampling to identify a subset of participants within each study group and by clinician. Researchers created a matrix of study groups and sequentially reviewed transcripts until they balanced the pre-specified study group. The researchers were blind to the content of the transcripts when they selected the transcripts to code, only looking at the study identifier and study group when selecting transcripts. In order to code at least one transcript from each surgeon, we coded all available transcripts for surgeons with fewer available recordings (i.e., <10). For surgeons with higher volumes, we randomly selected transcripts for analysis. The mean number of transcripts per surgeon was 11 (range 1–30). A total of 142 out of 311 transcripts were included. Two coders (VG, GE) reviewed transcripts line-by-line in NVivo 11 and ATLAS.ti. VG coded 100% of transcripts, while GE coded 20% of transcripts selected at random to ensure coding accuracy and consistency. The study team (MP, M-AD, RY) reviewed codes when questions or discrepancies arose and resolved disagreements. VG, MP, M-AD, RWY and GE had regular check-ins to discuss categories and codes.

First, we quantified (using frequency counts and percentages) the number of statements made within each of the categories in the Stivers and Barnes’ framework and our additional category about comparable options [7]. Next, we compared the number of statements made within each of the categories across study group (usual care vs. intervention groups) using chi-square analyses to compare percentages across groups. We modeled this approach on past work that has used this framework to explore the impact of the categories on patients’ medication decisions [28].

## Results

In the parent trial, of 2057 patients screened, 818 patients were eligible and approached; 622 consented to participate in the trial. Of sixteen surgeons, fourteen had audio-recorded encounters available for the parent analysis (two had low patient volume and/or consent procedures at their respective site that occurred after the encounter began). Recordings lasted 26 minutes on average (range 4–83 minutes).

Table 1 displays descriptive characteristics of the patient and surgeon participants in this analysis 142 of 311 surgeon-patient recorded encounters of 13 surgeons. We collected limited demographic information about surgeons to protect confidentiality. Patients were 59 years on average (range 25 to 89). Slightly more than half identified as White, non-Hispanic (81/142, 57.0%). About one-third (46/142; 32.4%) had a 4-year college degree. Surgeons were mostly female (10/13; 76.9%).

**Table 1:** Participant characteristics.

	Patient Participants (n = 142)				
Characteristic, n (%)	All Groups (n = 142)	Usual Care (n = 45)	Picture Option Grid (n = 64)	Option Grid (n = 33)	p-value^
Age, mean (SD)*	59.4 (11.1)	59.0 (10.7)	59.1 (12.0)	60.4 (10.1)	0.83
**Race, n (%)**					0.07
Asian	4 (2.8%)	1 (2.0%)	3 (5.0%)	0 (0.0%)	
Black, non-Hispanic	27 (19.0%)	8 (18.0%)	11 (17.0%)	8 (24.0%)	
Hispanic	20 (14.1%)	6 (13.0%)	13 (20.0%)	1 (3.0%)	
White, non-Hispanic	81 (57.0%)	25 (56.0%)	32 (50.0%)	24 (73.0%)	
Other	5 (3.5%)	4 (9.0%)	1 (2.0%)	0 (0.0%)	
Missing	5 (3.5%)	1 (2.0%)	4 (6.0%)	0 (0.0%)	
**Education, n (%)**					0.24
Never attended high school	5 (3.5%)	1 (2.0%)	3 (5.0%)	1 (3.0%)	
Some high school	17 (12.0%)	4 (9.0%)	10 (16.0%)	3 (9.0%)	
High school diploma or equivalent	32 (22.5%)	11 (24%)	9 (14.0%)	12 (36.0%)	
Some college	25 (17.6%)	9 (20.0%)	12 (19.0%)	4 (12.0%)	
Two-year degree	17 (12.0%)	3 (7.0%)	12 (19.0%)	2 (6.0%)	
Four-year degree	46 (32.4%)	17 (38.0%)	18 (28.0%)	11 (33.0%)	
**Surgery Choice, n (%)**					0.54
Lumpectomy	110 (77.5%)	35 (78.0%)	49 (77.0%)	26 (79.0%)	
Mastectomy	22 (15.5%)	7 (16.0%)	12 (19.0%)	3 (9.0%)	
Missing	10 (7.0%)	3 (7.0%)	3 (5.0%)	4 (12.0%)	
	**Surgeon Participants** (n = 13)				
**Group, n (%)**					
Option Grid	4 (31%)				
Picture Option Grid	5 (38%)				
Usual Care	4 (31%)				
**Female sex, n (%)**	10 (77%)				
**Years since graduating medical school, mean (range)**	24 years (10–44)				
**Years at current site, mean (range)**	11 years (<1 to 30)				
**Interest in SDM before trial**	11 (85%)				

^Chi-square analyses conducted to assess for statistical significance across the three trial groups for categorical characteristics. T-test used to assess for statistical significance for the one continuous characteristic (age).

*SD = standard deviation

Table 2 displays actions in the framework and examples of how decisions were discussed within each category of recommendations. Surgeons using one of the two conversation aids displayed more shared decision-making than those in usual care, describing options and reviewing potential harms and benefits by acknowledging clinical equipoise in all encounters (usual care 64% vs. intervention 100%; p<0.001). Nevertheless, surgeons across study groups gave recommendations about treatments that used authoritative *pronouncements* (usual care 51% vs. intervention 36%; p = .09) which does not align with shared decision-making principles.

**Table 2:** Representative quotations from coded transcripts of encounters.

Recommendation Action	Explanation	Frequency Across Groups	Example Quotes
**Pronouncement**	Clinician declares a treatment option and determines a care path, usually when no prior discussion of options has occurred. Sometimes happened before use of conversation aid in intervention groups.	Usual Care 51% Intervention Groups 36% χ2 = 2.87, p = .09	“If you can keep the breast, then keep it. It doesn’t make a difference in your overall survival.” (Usual Care, P126) “My recommendation for you would be for a minor surgery, which is to remove the lump.” (Usual Care, P78) “The first thing we do is surgery. We do a small surgery to remove just the tumor. I’m not cutting off your whole breast.” (Picture Option Grid, P52)
**Suggestion**	Prior to eliciting patient preferences, surgeon makes a recommendation, but mentions that it is the patient’s choice and that the recommended treatment is “optional.”	Usual care 27% Intervention Groups 27% χ2 = 0.000, p = 0.98	“The alternative to [lumpectomy] would be a bigger surgery… but I think [mastectomy] it’s probably more than you need. So, unless it’s really your preference, I wouldn’t direct you towards that.” (Usual Care, P31) “You have such a small tumor, so this [lumpectomy] is likely the better choice instead of taking out the whole breast, but this is your decision.” (Option Grid, P40) “It’s a very personal choice and a lot of people come in with a preference one way or another, I will never talk them out of it. If you walked in and just said, ‘What do you think?’ Honestly, I think a mastectomy is a really big operation for something this small.” (Picture Option Grid, P10)
**Proposal**	Surgeon makes recommendation, but decision-making is treated as shared between the surgeon and patient	Usual Care 21% Intervention Groups 26%; χ2 = 0.44, p = 0.51	“It’s just so small that I think that you could have a lumpectomy and do just as well with adjuvant chemotherapy… but, we can talk more about it, the pros and cons of it.” (Option Grid, P85) “Now, let me just tell you. You can do whatever you want but if you were my sister and that’s how I’m going to treat you … They’ll remove the area. Hopefully, get good margins… If you have a good result, then that’s great. You’ve saved yourself a big operation. No breast reconstruction and the same survival. That’s my opinion… So, you’re thinking lumpectomy? I am too.” (Option Grid, P19)
**Offer**	Surgeon presents an option as if the patient has initiated it. Often after patient preferences have been elicited. Often conflicts with surgeon’s stated preference.	Usual Care 40% Intervention Groups 40%; χ2 = .001, p = 0.98	“So, it sounds like you’re leaning towards the lumpectomy with the sentinel node… I think that makes sense. Good.” (Option Grid, P23) “Like I said, I’ll go along with whatever you want in the final analysis. I just want to make sure you’re well-informed about everything… I want you to have the most information so you can do the right thing for you…I’ll do whatever you want me to do, okay?” (Usual Care, P21) “If it’s a small tumor…it’s fine just to do this [lumpectomy]. . .Ultimately, the choice is yours. If you say, ‘I really, really want you to remove my breast. I’m just so nervous, just remove the whole thing,’ I would do that.” (Picture Option Grid, P118)
**Assertion**	Surgeon describes treatment benefit or drawback for patient without explicitly stating that the patient will receive that treatment. Can be informational, or can be used to guide or advise on choice if followed by a strong statement or recommendation.	Usual Care 71% Intervention Groups 66% χ2 = 0.37; p = 0.54	“We can do a lumpectomy instead of having to do a mastectomy, which of course most patients would prefer.” (Usual Care, P80) “Removing a whole breast is a bit more aggressive, requires more recovery time, and we really [only] do that if a patient has a really big tumor.” (Picture Option Grid, P93) “Some women say to me, ‘The idea of having any kind of defect or divot in my breast, I don’t want that. I’d much rather just have the breast off and reconstruction.’ (Option Grid, P50) “In order to avoid radiation, some patients say, ‘Just do a mastectomy.’” (Picture Option Grid, P87)
**Offer Comparable Options**	Emphasizes equipoise and includes a balanced comparison of options. Often facilitated by one of the conversation aids.	Usual Care 64% Intervention Groups 100%; X2 = 38.9; p<0.001	Alright, and so the type of surgery you choose depends largely upon what you prefer because you qualify by all other standards, okay? We’re going to talk about both of them and then you tell me what you want to do. Alright, so this chart *[uses conversation aid*] basically tells the difference between a lumpectomy and mastectomy. Alright, we’re going to go through it and if you come up with any questions, feel free to write on this. You come up with any questions, just ask them okay?” (Option Grid, P74) Surgeon: “It’s a personal decision. It’s a hard decision… They’ve done all of these studies, randomizing women to either one, and they followed them for 20 years. There’s no difference in outcome.” Patient: “So, I’m going to live either way?” Surgeon: “Exactly.” (Picture Option Grid, P6)

### Pronouncements and suggestions

Some surgeons made strong recommendations that could influence decision-making. These strong recommendations occurred in the *pronouncement* and *suggestion* categories of actions.

#### Pronouncements.

Pronouncements (the most authoritative action according to the taxonomy) were most common in the usual care group, but were present in all study groups. When surgeons made strong pronouncements, these pronouncements often came early in encounters, before patients had an opportunity to express their views or preferences.

For example, in the usual care group, one surgeon asserted early in the encounter, *“My recommendation for you would be for a minor surgery*, *which is to remove the lump” (Usual Care*, *P78)* without mentioning the alternative option, mastectomy, or seeking the patient’s perspective.

Another surgeon from the usual care group, told a nurse within earshot of the patient, *“we’re going to do a right lumpectomy on her*,*”* (*Usual Care*, *P27*) before discussing the treatment decision with the patient.

Even within the intervention groups, which included training in shared decision making and how to elicit patients’ preferences using a conversation aid, surgeons made pronouncements in 36% of clinical visits. For example, before reviewing the conversation aid, a surgeon in one of the intervention groups presented breast-conserving surgery with radiation as the default option to a patient, mentioning mastectomy only as a last resort.

*“The first thing we do is surgery. We do a small surgery to remove just the tumor. I’m not cutting off your whole breast. Your cancer is less than one centimeter. Super small. I make a small cut on the breast, remove it. It’s a same day surgery. You go home the same day… I told you about surgery and I said that we can remove just the tumor. Another option is to remove the whole breast. That’s [only done if it’s] a necessity…” (Picture Option Grid, P52)*

In a minority of these pronouncement scenarios, surgeons in the intervention groups did not use the conversation aid. Many also used the conversation aid to emphasize the choice they might recommend, highlighting benefits. For example, after a patient indicated she might choose breast-conserving surgery, the surgeon replied, “*All right*. *Good deal*. *So this [Option Grid] is just in my packet…it’s even in chart form side by side*, *all right*? *Let’s talk about radiation really quickly since you opted for the lumpectomy*. *It will likely be recommended because you chose a lumpectomy*.*” (Option Grid*, *P245)*.

#### Suggestions.

At times, surgeons’ recommendations were less emphatic, where the surgeon made *suggestions* to the patient using language that acknowledged patient choice. These suggestions happened in both the usual care (27%) as well as in the intervention groups (27%).

Sometimes surgeons used language like, *“this is your decision”* or *“this is your choice*,*”* or offered a brief review of the conversation aids while giving recommendations. In these cases, surgeons often moved quickly through the content of the conversation aids, without engaging the patients in complete discussions about the options or eliciting preferences.

In addition, sometimes contradictory concepts–*the acknowledgement of choice* as well as a strong *suggestion*–were combined:

*“I’ll be honest, it’s a very personal choice and a lot of people come in with a preference one way or another. I will never talk them out of it. [But] If you walked in and just said, ‘This was way more choice than I’m ready to make right now. What do you think?’ Honestly, I think a mastectomy is a really big operation for something this small. Truly. If you were really on the fence, which I don’t think you are, but I’m assuming anyway, then I would push you towards breast conservation. I just think it’s [mastectomy] a lot more surgery than you need.” (Picture Option Grid, P10)*

In other cases, the surgeons used the conversation aid as a tool to guide patients towards the surgeon’s own preference, most commonly breast-conserving surgery with radiation. For instance, they would mention that breast-conserving surgery is a smaller surgery, but not address radiation and related side effects, treatment burden, or risk of reoperation.

Without explicit guidance in the role patients could play in decision-making, many patients shared a preference for a larger surgery, but were careful to allow the surgeon input, which surgeons sometimes used to propose a less invasive surgical plan.

*Patient: “So I think I still want the bilateral [mastectomy] because I don’t really want the lumpectomy and radiation due to [family history]–we can talk about it, but if you think I’m totally crazy, you can say ‘Think about it’ and I will. I know it’s a small cancer.”*

*Surgeon: “Well, I want you to think about, I think this is something that we can take good care of very easily [with a lumpectomy]…”*

(*Usual Care*, *P21*)

### Proposals, offers, and presenting comparable options

Some surgeons made recommendations that complemented shared decision-making principles of patient collaboration and the importance of patients’ preferences by making *proposals* (proposals (usual care 21% vs. intervention 26%; p = 0.51) and *offers* (offers (usual care 40% vs. intervention 40%; p = 0.98). Every clinician in the intervention group *presented comparable options*, consistent with shared decision-making (usual care 64% vs intervention 100%; p<0.001).

#### Proposals.

Some surgeons made recommendations and emphasized that decision-making was a shared process. Proposals were often made in the context of patient preferences and paired with statements such as: *“if you’re interested in”* or *“I want you to think about it*.” They sometimes mentioned patient priorities, like getting back to work, during these proposal recommendations.

One surgeon in an intervention group acknowledged the patient’s potential choice, though used a strong recommendation about what the surgeon would hypothetically say to his/her ‘sister’:

*“Now, let me just tell you. You can do whatever you want, but if you were my sister and that’s how I’m going to treat you [I suggest lumpectomy]… They’ll remove the area. Hopefully, get good margins… If you have a good result, then that’s great. You’ve saved yourself a big operation. No breast reconstruction and the same survival. That’s my opinion…you’re thinking lumpectomy? I am too.” (Option Grid, P19)*.

The surgeon offered a recommendation, while inviting the patient to agree. In this case, the patient’s treatment preference was not yet known or explored.

Sometimes the interventions themselves helped move an interaction from *suggestion* to *proposal*, with surgeons offering their recommendations before or after reviewing the conversation aid. One surgeon said,

*“It’s [the tumor] just so small that I think that you could have a lumpectomy and do just as well with adjuvant chemotherapy… but, we can talk more about it, the pros and cons of it.” (Option Grid, P85)*

In this case, the surgeon offered a recommendation, followed by the possibility of more talk, using the conversation aid.

#### Offers & offering comparable options.

Offers occurred across intervention and usual care groups. A few surgeons in the usual care group engaged in deliberation with patients, demonstrating that presenting options were not necessarily contingent on tools like conversation aids. One surgeon said, “*I want you to have the most information so you can do the right thing for you*… *I’ll do whatever you want me to do*, *okay*?*” (P21*, *Usual Care)*. In this case, the surgeon let the patient’s preferences lead to a decision to plan a mastectomy. Surgeons in the usual care group, however, were less likely to offer comparable options compared to surgeons using a conversation aid (offers comparable options (usual care 64.4% vs intervention 100%; p<0.001).

In the intervention groups, the conversation aids sometimes facilitated *offers*. For example, as a patient reviewed the conversation aid, her surgeon asked her which surgery she was leaning towards. She pointed to the breast-conserving surgery with radiation option, and her surgeon affirmed her choice. The surgeon only offered a recommendation in the context of affirming the patient’s decision, saying, *“that makes the most sense to me as well*.*” (P40*, *Option Grid)*. Similarly, one patient expressed an early preference for mastectomy. Her surgeon validated her choice, even while expressing a preference herself for lumpectomy, *“If it’s a small tumor…it’s fine just to do this [lumpectomy]*…*[but] ultimately*, *the choice is yours*. *If you say*, *‘I really*, *really want you to remove my breast*. *I’m just so nervous*, *just remove the whole thing*,*’ I would do that*.*” (Picture Option Grid*, *P118)*. In this case, the surgeon responded to the patient’s preferences and *offered* both choices as valid treatment options.

When surgeons used the tools as intended, with fidelity to the training provided in the trial, they were more inclined to *offer comparable options*. One surgeon presented the conversation aid and then told the patient, “*basically there are two types of surgeries I can offer you and I think you’re a candidate for either one*.*” (Option Grid*, *P74)*. Another patient struggled to decide between surgery options. Her surgeon talked to her about both options, using the conversation aid, without recommending one surgery or the other. She also affirmed the patients’ feelings, noting that it was a difficult decision:

*“So, let’s use our sheet…There are two options with surgery… there’s no difference in outcome, in terms of it coming back and your overall survival…there are benefits to both. A lumpectomy is a same day surgery. You’re in and out in the same day. It’s quick. You can just get it out, and then you can start the chemo, and start to go through the treatment. [With a mastectomy], if we remove the breast, we can do reconstruction. Then, you don’t have to be worried, “Is it going to come back? Is it going to come back?” It’s a personal decision. It’s a hard decision.” (Picture Option Grid, P6)*.

#### Assertions were often followed by pronouncements.

Assertions (providing information about options) occurred across all study groups (assertions usual care 71% vs. intervention 66%; p = 0.54). Some were simply informational, while others were used to encourage patients toward an option with clear *pronouncements* following the information. For example, in the usual care group, one surgeon told a patient, *“If it [additional biopsy] comes back negative*, *then you’re an excellent candidate to have breast-conserving surgery…”* The assertion was then followed by a strong *pronouncement*: “*Okay*? *That’s what you need to have done*.*” (Usual Care*, *P60)*.

Similarly, when reviewing the intervention, one surgeon made an *assertion* by saying, *“Removing a whole breast is a bit more aggressive*, *requires more recovery time*,*”* followed by a strong *pronouncement*, *“and we really [only] do that [mastectomy] if a patient has a really big tumor*. *In your case*, *it will be safe to just remove the tumor*.*” (Picture Option Grid*, *P93)*.

## Discussion

In our analysis, many surgeons made authoritative recommendations within the categories of *pronouncements* and *suggestions* to patients with early-stage breast cancer making a preference-sensitive surgery choice. Those trained in shared decision-making and provided with a conversation aid were more likely than those practicing as usual to describe clinical equipoise and support deliberation during the encounter, describing this important concept in 100% of encounters in the intervention groups. However, across study groups, many surgeons used informational *assertions* to support strong *pronouncements* about what to choose. This study suggests that presenting comparable options does not necessarily lead to shared decision-making without support for collaborative partnership and consistent language acknowledging choice [5, 29].

Our study reinforces the notion that shared decision-making is nuanced and complex in the clinical environment. Elwyn and colleagues’ Three Talk Model of shared decision-making emphasizes Team Talk, where clinician and patient establish a partnership, Option Talk, where they discuss alternatives and risks, and Decision Talk, where the discussion turns to the patients’ preferences to reach an agreed-upon treatment plan [27]. Part of this shared decision making process can occur with or without true clinical equipoise. In fact, decision aids often support Option Talk, as they help patients deliberate and clarify their preferences through understanding information about options and thinking about how possible outcomes of options might impact their life [30, 31]. Likewise, the conversation aids in this study usually prompted surgeons to perform Option Talk, as they discussed risk and benefit information for each surgical choice. However, full shared decision making in the presence of more than one reasonable option should engage patients as partners through making this choice, exploring patient preferences rather than guiding the decision based on a clinician’s preference. As surgeons moved along in the consultation about breast cancer surgery choice, subtle or explicit recommendations were sometimes inconsistent with Team Talk and Decision Talk. *Pronouncements* (authoritative statements) in the intervention groups sometimes included Option Talk, but not Team Talk or Decision Talk. When Team Talk occurred with *suggestions* (more subtle but strong recommendations), it was often followed by strong endorsements of one surgical choice over another, suggesting that clinicians might not have perceived true equipoise about the decision. Even in *proposals* and *offers* that acknowledged patient autonomy while providing advice, surgeons occasionally oscillated between engaging in a deliberation and making recommendations. They often adapted their approach to shared decision-making as clinical visits progressed.

Given the complexity of practicing shared decision-making, even with training and tools, surgeons might need more support for how to elicit patients’ preferences and align care with those preferences before endorsing an option. Skills like active listening and responding to patient preferences might need to be paired with shared decision-making interventions. Most, if not all, surgeons in the study displayed humanistic elements of shared decision-making, like respect, compassion and empathy [32]. However, within their patient-centered approach, their own personal preferences came through either explicitly or subtly. Because of the natural tendency to provide recommendations, support in making recommendations *after* responding to patients’ preferences could enhance shared decision-making training. Training might need to include ways to respond to patients’ questions such as “what would you do?” while remaining consistent with the spirit of shared decision-making [33]. Training could include more specific role-playing about how to make a recommendation after engaging with patients to explore preferences (i.e., during Decision Talk), not at the start of an encounter or during an explanation of the pros and cons of options (i.e., during Option Talk). Finally, national organizations that might encourage surgeons to reduce mastectomy rates could instead support surgeons in documenting when they have engaged in shared decision-making to support patients’ informed, preference-consistent choices.

The *What Matters Most* trial provided a unique opportunity to explore the role that clinician communication styles play in surgical encounters [10] by providing shared decision-making training (including in communicating about comparable options, facilitating patient engagement and encouraging deliberation), tools and support to clinicians [2, 34]. Using the established communication framework about treatment recommendations was novel in the context of shared decision making research, and coding audio-recorded encounters built on this framework of recommendations to examine results. Most past work examining shared decision making behaviors during clinical consultations has explored whether clinicians acknowledge options and invite the patient to participate [35–38], rather than whether and how they make treatment recommendations. More recently, scholars recommend focusing on care that works for patients and makes sense given the context of their lives, above and beyond describing information about clinical outcomes [39]. The research presented here adds to this approach about how information is integrated into clinical encounters when formulating treatment plans and weighing reasonable options.

However, there are some study limitations that should be considered. These analyses were conducted across 142 patients of 13 surgeons discussing early-stage breast cancer surgery. Results might not apply across other contexts outside of surgery or breast cancer conversations. It is possible that clinicians did not support the overall shared decision-making model as they were faced with specific clinical scenarios or patient characteristics [3]. They might also believe that individual patients could benefit more from one treatment given baseline risk, age, or comorbidities. They might feel pressure from national societies to reduce the use of mastectomies when breast-conserving surgery with radiation is an option, if women mistakenly think a larger surgery will improve survival [14–16]. Thus, the surgeons might not have perceived true equipoise across surgery options. However, patients’ eligibility for both surgical options was confirmed by surgeons prior to inclusion. Almost all surgeons were interested in shared decision-making before the study started. An in-depth discourse analysis [40, 41] could provide more details on the quality of conversations, rather than focusing specifically on treatment recommendations and/or conversation descriptions.

## Conclusion

Our study suggests that clinicians often make both subtle and explicit recommendations about choices. Conversation aids can facilitate offers of comparable options, but do not on their own support shared decision-making without an explicit acknowledgement of equipoise and an invitation to collaborate. More work should explore how language and framing of recommendations can influence choices.

## Supporting information

S1 Appendix Appendix A displays the Option Grid and Picture Option Grid used in the study. (DOCX)

S2 Appendix Appendix B displays the codebook used for analysis. (DOCX)

## References

[1] Elwyn G, Edwards A, Kinnersley P, Grol R. Shared decision making and the concept of equipoise: the competences of involving patients in healthcare choices. The British journal of general practice: the journal of the Royal College of General Practitioners. 2000;50(460):892–9. 11141876 PMC1313854

[2] Joseph-Williams N, Lloyd A, Edwards A, Stobbart L, Tomson D, Macphail S, et al. Implementing shared decision making in the NHS: lessons from the MAGIC programme. Bmj. 2017;357:j2005. doi: 10.1136/bmj.j2005 28420639 PMC6284240

[3] Pollard S, Bansback N, Bryan S. Physician attitudes toward shared decision making: A systematic review. Patient Educ Couns. 2015;98(9):1046–57. doi: 10.1016/j.pec.2015.05.004 26138158

[4] Mishra MK, Saunders CH, Rodriguez HP, Shortell SM, Fisher E, Elwyn G. How do healthcare professionals working in accountable care organisations understand patient activation and engagement? Qualitative interviews across two time points. BMJ Open. 2018;8(10):e023068. doi: 10.1136/bmjopen-2018-023068 30385443 PMC6252703

[5] Thomas EC, Bass SB, Siminoff LA. Beyond rationality: Expanding the practice of shared decision making in modern medicine. Social Science & Medicine. 2021;277:113900. doi: 10.1016/j.socscimed.2021.113900 33838448 PMC8119352

[6] Landmark AM, Svennevig J, Gulbrandsen P. Negotiating treatment preferences: Physicians’ formulations of patients’ stance. Soc Sci Med. 2016;149:26–36. doi: 10.1016/j.socscimed.2015.11.035 26699275

[7] Stivers T, Barnes RK. Treatment Recommendation Actions, Contingencies, and Responses: An Introduction. Health Communication. 2018;33(11):1331–4. doi: 10.1080/10410236.2017.1350914 28825505

[8] Maskrey N. Shared decision making: why the slow progress? An essay by Neal Maskrey. BMJ. 2019;367:l6762. doi: 10.1136/bmj.l6762 31806646

[9] Durand MA, Yen RW, O’Malley AJ, Politi MC, Dhage S, Rosenkranz K, et al. What matters most: protocol for a randomized controlled trial of breast cancer surgery encounter decision aids across socioeconomic strata. BMC Public Health. 2018;18(1):241. doi: 10.1186/s12889-018-5109-2 29439691 PMC5812033

[10] Durand MA, Yen RW, O’Malley AJ, Schubbe D, Politi MC, Saunders CH, et al. What matters most: Randomized controlled trial of breast cancer surgery conversation aids across socioeconomic strata. Cancer. 2021;127(3):422–36. doi: 10.1002/cncr.33248 33170506 PMC7983934

[11] Morrow M, Strom EA, Bassett LW, Dershaw DD, Fowble B, Giuliano A, et al. Standard for breast conservation therapy in the management of invasive breast carcinoma. CA Cancer J Clin. 2002;52(5):277–300. doi: 10.3322/canjclin.52.5.277 12363326

[12] Tung NM, Boughey JC, Pierce LJ, Robson ME, Bedrosian I, Dietz JR, et al. Management of Hereditary Breast Cancer: American Society of Clinical Oncology, American Society for Radiation Oncology, and Society of Surgical Oncology Guideline. Journal of clinical oncology: official journal of the American Society of Clinical Oncology. 2020;38(18):2080–106. 32243226 10.1200/JCO.20.00299

[13] National Cancer Institute. Breast Cancer Treatment (Adult) (PDQ®)–Patient Version 2021 [updated January 8, 2021. Available from: https://www.cancer.gov/types/breast/patient/breast-treatment-pdq#link/_229_toc

[14] National Cancer Institute. NIH consensus conference. Treatment of early-stage breast cancer. Jama. 1991;265(3):391–5. 1984541

[15] American Society of Breast Surgeons. Five Things Physicians and Patients Should Question 2019 [updated 2019. Available from: https://www.choosingwisely.org/societies/american-society-of-breast-surgeons/

[16] Covelli AM, Baxter NN, Fitch MI, McCready DR, Wright FC. ’Taking control of cancer’: understanding women’s choice for mastectomy. Ann Surg Oncol. 2015;22(2):383–91. doi: 10.1245/s10434-014-4033-7 25190120

[17] Cronin PA, Olcese C, Patil S, Morrow M, Van Zee KJ. Impact of Age on Risk of Recurrence of Ductal Carcinoma In Situ: Outcomes of 2996 Women Treated with Breast-Conserving Surgery Over 30 Years. Ann Surg Oncol. 2016;23(9):2816–24. doi: 10.1245/s10434-016-5249-5 27198513 PMC4995886

[18] Braunstein LZ, Taghian AG, Niemierko A, Salama L, Capuco A, Bellon JR, et al. Breast-cancer subtype, age, and lymph node status as predictors of local recurrence following breast-conserving therapy. Breast Cancer Research and Treatment. 2017;161(1):173–9. doi: 10.1007/s10549-016-4031-5 27807809

[19] Vos E, Koppert L, van Lankeren W, Verhoef C, Koerkamp BG, Hunink M. A preliminary prediction model for potentially guiding patient choices between breast conserving surgery and mastectomy in early breast cancer patients; a Dutch experience. Qual Life Res. 2018;27(2):545–53. doi: 10.1007/s11136-017-1740-0 29147887 PMC5846961

[20] Lagendijk M, van Egdom LSE, van Veen FEE, Vos EL, Mureau MAM, van Leeuwen N, et al. Patient-Reported Outcome Measures May Add Value in Breast Cancer Surgery. Annals of Surgical Oncology. 2018;25(12):3563–71. doi: 10.1245/s10434-018-6729-6 30178391

[21] Palve JS, Luukkaala TH, Kääriäinen MT. Predictive risk factors of complications in different breast reconstruction methods. Breast Cancer Res Treat. 2020;182(2):345–54. doi: 10.1007/s10549-020-05705-3 32468337 PMC7297836

[22] Hansen N, Espino S, Blough JT, Vu MM, Fine NA, Kim JYS. Evaluating Mastectomy Skin Flap Necrosis in the Extended Breast Reconstruction Risk Assessment Score for 1-Year Prediction of Prosthetic Reconstruction Outcomes. Journal of the American College of Surgeons. 2018;227(1):96–104. doi: 10.1016/j.jamcollsurg.2018.05.003 29778821

[23] Myckatyn TM, Parikh RP, Lee C, Politi MC. Challenges and Solutions for the Implementation of Shared Decision-making in Breast Reconstruction. Plastic and reconstructive surgery Global open. 2020;8(2):e2645–e. doi: 10.1097/GOX.0000000000002645 32309090 PMC7159965

[24] Freeman AB, Huang B, Dragun AE. Patterns of care with regard to surgical choice and application of adjuvant radiation therapy for preinvasive and early stage breast cancer in rural Appalachia. Am J Clin Oncol. 2012;35(4):358–63. doi: 10.1097/COC.0b013e3182118d27 21422902

[25] Gu J, Groot G. Creation of a new clinical framework–why women choose mastectomy versus breast conserving therapy. BMC Med Res Methodol. 2018;18(1):77. doi: 10.1186/s12874-018-0533-7 29986654 PMC6038174

[26] Mac Bride MB, Neal L, Dilaveri CA, Sandhu NP, Hieken TJ, Ghosh K, et al. Factors associated with surgical decision making in women with early-stage breast cancer: a literature review. J Womens Health (Larchmt). 2013;22(3):236–42. doi: 10.1089/jwh.2012.3969 23428286

[27] Elwyn G, Durand MA, Song J, Aarts J, Barr PJ, Berger Z, et al. A three-talk model for shared decision making: multistage consultation process. BMJ. 2017;359:j4891. doi: 10.1136/bmj.j4891 29109079 PMC5683042

[28] Thompson L, McCabe R. How Psychiatrists Recommend Treatment and Its Relationship with Patient Uptake. Health Communication. 2018;33(11):1345–54. doi: 10.1080/10410236.2017.1350916 28812368 PMC6068540

[29] Montori VM, Kunneman M, Brito JP. Shared decision making and improving health care: The answer is not in. JAMA. 2017;318(7):617–8. doi: 10.1001/jama.2017.10168 28810005

[30] Elwyn G, Frosch D, Rollnick S. Dual equipoise shared decision making: Definitions for decision and behaviour support interventions. Implementation Science. 2009;4:75. doi: 10.1186/1748-5908-4-75 19922647 PMC2784743

[31] Stacey D, Legare F, Lewis K, Barry MJ, Bennett CL, Eden KB, et al. Decision aids for people facing health treatment or screening decisions (Cochrane Review). Cochrane Database of Systematic Reviews. 2017(4):Art. No.: CD001431. 10.1002/14651858.CD001431.pub5 PMC6478132 28402085

[32] Gysels M, Richardson A, Higginson IJ. Communication training for health professionals who care for patients with cancer: a systematic review of effectiveness. Supportive Care in Cancer. 2004;12(10):692–700. doi: 10.1007/s00520-004-0666-6 15258839

[33] Politi MC, Dizon DS, Frosch DL, Kuzemchak MD, Stiggelbout AM. Importance of clarifying patients’ desired role in shared decision making to match their level of engagement with their preferences. BMJ: British Medical Journal. 2013;347. doi: 10.1136/bmj.f7066 24297974

[34] Legare F, Ratte S, Gravel K, Graham ID. Barriers and facilitators to implementing shared decision-making in clinical practice: update of a systematic review of health professionals’ perceptions. Patient Educ Couns. 2008;73(3):526–35. doi: 10.1016/j.pec.2008.07.018 18752915

[35] Couët N, Desroches S, Robitaille H, Vaillancourt H, Leblanc A, Turcotte S, et al. Assessments of the extent to which health-care providers involve patients in decision making: a systematic review of studies using the OPTION instrument. Health expectations: an international journal of public participation in health care and health policy. 2015;18(4):542–61. doi: 10.1111/hex.12054 23451939 PMC5060794

[36] Barr PJ, Forcino RC, Thompson R, Ozanne EM, Arend R, Castaldo MG, et al. Evaluating CollaboRATE in a clinical setting: analysis of mode effects on scores, response rates and costs of data collection. BMJ Open. 2017;7(3):e014681. doi: 10.1136/bmjopen-2016-014681 28341691 PMC5372080

[37] Forcino RC, Barr PJ, O’Malley AJ, Arend R, Castaldo MG, Ozanne EM, et al. Using CollaboRATE, a brief patient-reported measure of shared decision making: Results from three clinical settings in the United States. Health expectations: an international journal of public participation in health care and health policy. 2018;21(1):82–9. doi: 10.1111/hex.12588 28678426 PMC5750739

[38] Forcino RC, Thygeson M, O’Malley AJ, Meinders MJ, Westert GP, Elwyn G. Do collaboRATE Scores Reflect Differences in Perceived Shared Decision-Making Across Diverse Patient Populations? Evidence From a Large-Scale Patient Experience Survey in the United States. Journal of patient experience. 2020;7(5):778–87. doi: 10.1177/2374373519891039 33294615 PMC7705838

[39] Kunneman M, Hargraves IG, Sivly AL, Branda ME, LaVecchia CM, Labrie NHM, et al. Co-creating sensible care plans using shared decision making: Patients’ reflections and observations of encounters. Patient education and counseling. 2021. doi: 10.1016/j.pec.2021.10.003 34711446

[40] Stivers T, Timmermans S. Medical Authority under Siege: How Clinicians Transform Patient Resistance into Acceptance. J Health Soc Behav. 2020;61(1):60–78. doi: 10.1177/0022146520902740 32073304

[41] Barnes RK. Conversation Analysis of Communication in Medical Care: Description and Beyond. Research on Language and Social Interaction. 2019;52(3):300–15.

