# Supplementary figures and images for: An absence of equipoise: Examining surgeons’ decision talk during encounters with women considering breast cancer surgery
Source: PLoS One. 2021 Dec 16;16(12):e0260704. doi: 10.1371/journal.pone.0260704 (PMC8675712; doi:10.1371/journal.pone.0260704)

Appendix A. Conversation Aids: Option Grid (left) and Picture Option Grid Sample Page (right)


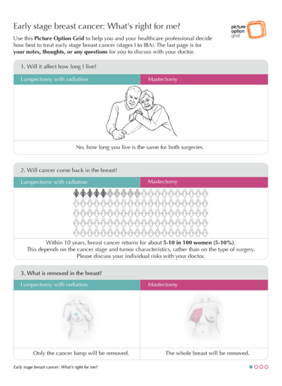

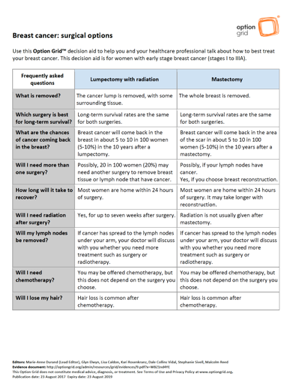

Supplement: S1 Appendix — (DOCX) [file pone.0260704.s001.docx]
