# Supplementary material for: An absence of equipoise: Examining surgeons’ decision talk during encounters with women considering breast cancer surgery
Source: PLoS One. 2021 Dec 16;16(12):e0260704. doi: 10.1371/journal.pone.0260704 (PMC8675712; doi:10.1371/journal.pone.0260704)
Supplement: S2 Appendix — (DOCX) [file pone.0260704.s002.docx]

Appendix B. Codebook

| **Coding Dimension** | **Options** | **Explanation** | **Examples** |
| --- | --- | --- | --- |
| Social action | **Pronouncement** | Physician asserts recommendation as instigator, decision maker and presents as already determined. | “I recommend a lumpectomy” “You’re a great candidate for breast conserving surgery. Here’s what we’ll do… That’s what you need to have done.” |
|  | **Suggestion** | Physician recommends as instigator but treats patient as decision maker and medication as optional. Comparable options may be implied but no deliberation invited or expression of equipoise made. | “You could try a lumpectomy.” “At your age, you’d probably want reconstruction.” “Usually for patients like you with a mutation we recommend a bilateral mastectomy, but if you don’t want that we can watch more closely.” “It looks like lumpectomy would be very effective for you. Is that the option you were hoping for?” “It’s up to you, but you don’t have to lose your breast.” |
|  | Proposal | Physician recommends as instigator but decision making is treated as shared by doctor and patient. Proposals highlight the recommendation as speculative. Two options may be mentioned, but no expression of equipoise. Deliberation not invited. | “Let’s try a lumpectomy” “I don’t see any reason you need a mastectomy.” “I think we could do a lumpectomy.” |
|  | Offer | Physician treats patient as having instigated recommendation and as the decision maker.  Two options may be mentioned, but no expression of equipoise. Two options are not proposed. Deliberation not invited. | “I’d be willing to let you try a lumpectomy, but that would not be my first choice for you”... “Based on all the preferences you just told me about, what I would recommend for you would be lumpectomy.” |
|  | Assertion | Physician asserts a generalization about a treatment’s benefit implying a recommendation but not proffering an overt directive. Two options are not proposed. Deliberation not invited. | “You are a good candidate for a lumpectomy.” |
| (Added) | Offer comparable options | Physician asserts that there is more than one option and both are reasonable. Comparison of options proposed. Deliberation invited. | “You could either choose a lumpectomy or a mastectomy.” |
| Strength of Endorsement (added) | Weak / Moderate | The recommendation is treated as ranging from likely to improve the patient’s health to of only marginal value to the patient | Pronouncement: “I’d like to try…”  Suggestion: “You might want to  think about …”  Proposal: “We could try …”  Offer: “I’d be willing to let you try  X”  Assertion: “Some of my patients  have felt that X was right for them.” |
|  | Strong | The recommendation is treated as a necessity or as very important | Pronouncement: “I’m gonna start  you on…”  Suggestion: “You really ought to  try …”  Proposal: “Shall we start on …”  Offer: “I could give you …”  Assertion: “Most people find that  X is a good fit.” |
| **Partnership Reference** |  | A reference to “we” or “us” that invokes the doctor and patient (but not the doctor and other professionals or the institution) | “Let’s see how it goes” “I’m going to treat you like you’re my sister so I want you to have the very best.” |
| **Patient uptake** |  |  |  |
|  | None | The patient does nothing in response to the recommendation. | — |
|  | Acknowledgment | The patient’s response is not clearly understandable as acceptance but does receipt the physician’s turn. | Uh huh, Mm hm, “So many decisions.” “Ok” |
|  | Acceptance | The patient vocally accepts the physician’s recommendation. | Okay, That’s fine. That sounds like a good plan. |
|  | Resistance | The patient vocally resists the physician’s recommendation by questioning it, challenging it, countering, proposing, or requesting an alternative medication. | “I don’t need a mastectomy?”; “But I’m worried about my risk of recurrence.” |
| **Initiator** | Instigator / Initiator |  |  |
|  | Clinician instigates | The clinician starts the discussion about treatment choice, leading the conversation. Can be any category, from suggestion, offer of comparable options, pronouncement, etc. | “have you thought about what choice you might want?” or “you are an excellent candidate for a lumpectomy.” |
|  | Patient instigates | The patient starts the discussion about treatment choice, leading the conversation. | “I think I want a mastectomy.” |
|  | Family member or friend instigates | A family member or supportive other starts the discussion about treatment choice. | “She’s thought about it and is leaning toward a lumpectomy.” |
| **Time to deliberate** | Deliberation | The surgeon explicitly encourages the patient to take time to think through her options | “You have time to think this through. Let’s meet again/talk again after you think about these options.” “We can schedule now if you want, but if you want to consider all these options, you do not have to decide now and you can call me.” |
| **Patient preferences** | Statement | Patient clearly states preferences | “I’d rather live ten years playing tennis than have a stunted serve for the rest of my life.” |
|  | Incorporates | Surgeon incorporates patient’s preferences into discussion | “This surgery will not affect your tennis serve.” |
